# RankProdIt: A web-interactive Rank Products analysis tool

**DOI:** 10.1186/1756-0500-3-221

**Published:** 2010-08-06

**Authors:** Emma Laing, Colin P Smith

**Affiliations:** 1 Faculty of Health and Medical Sciences, University of Surrey, Guildford, Surrey, GU2 7XH, UK

## Abstract

**Background:**

The first objective of a DNA microarray experiment is typically to generate a list of genes or probes that are found to be differentially expressed or represented (in the case of comparative genomic hybridizations and/or copy number variation) between two conditions or strains. Rank Products analysis comprises a robust algorithm for deriving such lists from microarray experiments that comprise small numbers of replicates, for example, less than the number required for the commonly used t-test. Currently, users wishing to apply Rank Products analysis to their own microarray data sets have been restricted to the use of command line-based software which can limit its usage within the biological community.

**Findings:**

Here we have developed a web interface to existing Rank Products analysis tools allowing users to quickly process their data in an intuitive and step-wise manner to obtain the respective Rank Product or Rank Sum, probability of false prediction and *p*-values in a downloadable file.

**Conclusions:**

The online interactive Rank Products analysis tool RankProdIt, for analysis of any data set containing measurements for multiple replicated conditions, is available at: http://strep-microarray.sbs.surrey.ac.uk/RankProducts

## Findings

The identification of differentially expressed or represented entities (genes/probes) between two conditions or strains, respectively, in a DNA microarray experiment is often the first task following data normalisation. However, to identify such entities it is no longer considered acceptable to apply an arbitrary fold-change threshold above which the difference in transcriptional or presence/absence status of an entity is defined. Instead, confidence through a test-statistic is expected. Of the many statistical methods that exist (and must be chosen between) for calculating test-statistics in the microarray field most are variants of the t-test, either traditional of modified. Whilst these methods are powerful their use has been shown to be limited when applied to 'noisy' data sets: few (less than 10) biological replicates and/or a high degree of variability between biological replicates [1,2]. Rank Products analysis (and the similar approach of Rank Sum [3] analysis) is an algorithm with which a confidence value can be obtained and an alternative to those statistical methods that require many biological replicates with little variability; it is robust against the noise in a microarray experiment and still retains sensitivity [1-4].

To date users wishing to conduct Rank Products analysis on their own data set have had the options of 1) manually calculating the Rank Products and associated statistics, 2) using the R [5,6] package RankProd [7] or 3) running a Perl script on their own computer [8]. Clearly, option 1 is unsuitable due to the time it takes to prepare the data set, learn the protocol/algorithm and perform many calculations. Although option 2 avoids manual calculation it requires familiarity with R [5,6] which can be daunting to some biologists. Whilst option 3 is less demanding (only requiring a command line entry) the script is only able to take log ratios and not linear data, which may therefore require a re-scaling of data. Thus, it is apparent that there is a need for an interface to the Rank Products tool such that users can analyse their own data in an intuitive, 'clickable' manner. Here we present the first online interactive Rank Products analysis tool RankProdIt.

## Implementation

RankProdIt is a web interface developed in haXe [9] that calls Perl CGI scripts to upload the data file, generate the R [5,6] commands and execute R on the server in slave mode. For the Rank Products and Rank Sum analysis all user selected parameters are passed to the R package RankProd [7]. Note, that the default 100 permutations of RankProd, for calculating the probability of observing a Rank Product and/or Rank Sum by chance, for both Rank Products and Rank Sum analysis is retained in RankProdIt.

RankProdIt is a generic tool, able to accept any data set containing replicated samples for at least two conditions. Thus, whilst this manuscript documents RankProdIt for microarray data analysis, it can be applied to other high throughput data sets such as next-generation sequencing, proteomic and metabolomic data. Input measurements can either be in the form of absolute levels, where row-element *k *has measurements in multiple columns for each condition *i *and *j*, such as that obtained from single-colour microarray experiments, or in the form of ratios, where each column of row-element *k *is a ratio of conditions (*i*/*j*), as obtained from two-colour microarray experiments.

To process data using RankProdIt a user submits a tab-delimited text file that contains a row-identifier (typically gene/probe identifier) column and several columns containing data; missing data is represented by NA or NaN. The input file is not required to have columns in any particular order and columns containing data *not *to be used in the analysis can also be included. A header row does not need to be included but if so, there must only be one. An example input file is given in Additional file 1.

**Figure 1:**
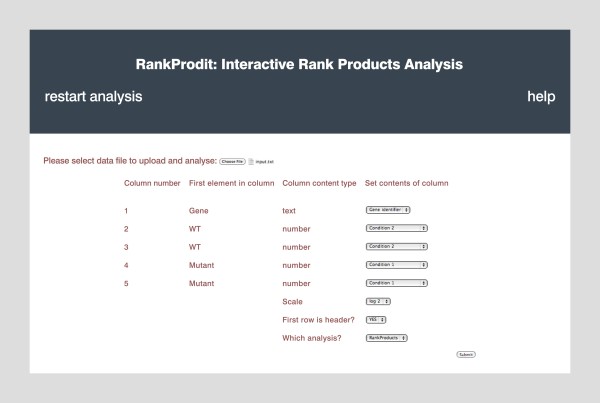
**An example page following successful submission of data**. The image depicts all fields that are required to be entered for Rank Products analysis of an uploaded input file and the output of the tool.

Once the input file is successfully checked and uploaded, for which there is constant progress feedback, a form containing a select box for each column in the file is produced; each select box denotes the classification of the column contents and how the column is to be handled in subsequent analysis. To aid the user RankProdIt attempts to predict the contents of each column and the initial selection of the select boxes reflects this. Still, the user can define how each column in the input file is to be handled (see Figure 1 for an example form given the input file in Additional file 1). Each column is readily identifiable through the column number (the order in which it appears in the input file) and associated information about that column (whether it contains text or numbers and the first element in the column) given in the form. A column can be selected to be either: a gene (row) identifier, ignored, a condition 1 or condition 2 sample (for absolute level based data), or a condition1/condition2 or condition2/condition1 sample (for ratio based data). For successful submission and correct execution of Rank Products or Rank Sum analysis a user must select only one column as a gene identifier and either:

- at least two columns as condition 1 samples *and *at least two columns as condition 2 samples

or

- at least two columns as condition1/condition2 *or *condition2/condition1 samples

If the correct selections are not made an error message is given following submission. Note, that whilst it is possible to perform Rank Products and/or Rank Sum analysis with as few as two biological replicates for each condition, it is recommended that a greater number of replicates be provided for greater confidence in data reliability.

The scale of the input data and the presence of a column header row is automatically selected by RankProdIt. Prior to submission the user can select whether to perform Rank Products or Rank Sum analysis; by default Rank Products analysis is selected.

Upon successful submission the data selected by the user is imported into R and Rank Products or Rank Sum analysis is performed by the RankProd package [7]; whilst the Rank Products/Sum analysis is being conducted an indication of processing is given, alerting the user that the analysis has not finished. If the data and selections made by the user do not cause an error within the RankProd [7] package a link to the output file is provided, for the user to download the results.

An example of an output (results) file is given in Additional file 2 and a brief description of columns within an output file is provided in Additional file 3. The output tab-delimited text file of RankProdIt is suitable to open with any spreadsheet software for data interpretation and/or further analysis (e.g. the enhanced distribution calculations for Rank Products that can easily be calculated in Excel [10]).

## Availability and requirements

**Project name: **RankProdIt

**Project home page: **http://strep-microarray.sbs.surrey.ac.uk/RankProducts/

**Any restrictions to use by non-academics: **None

## Competing interests

The authors declare that they have no competing interests.

## Authors' contributions

EL designed and developed the web tool. EL and CPS conceived the project and wrote the manuscript.

## Supplementary Material

Additional file 1 **An example input file**. An example data set representative of absolute level measurement data. Click here for file

Additional file 2 **An example output file**. The RankProdIt output file generated from submitting Additional File 1 to RankProdIt for Rank Products analysis. Click here for file

Additional file 3 **Description of columns in an output file**. Gives a description of the contents of columns in a RankProdIt output file. Click here for file

## References

[B1] Jeffery IB Desmond GH Culhane AC Comparison and evaluation of methods for generating differentially expressed gene lists from microarray data BMC Bioinformatics 2006 7 359 10.1186/1471-2105-7-359 16872483 PMC1544358

[B2] Hong F Breitling R A comparison of meta-analysis methods for detecting differentially expressed genes in microarray experiments Bioinformatics 2008 24 374 382 10.1093/bioinformatics/btm620 18204063

[B3] Breitling R Herzyk P Rank-based methods as a non-parametric alternative of the T-statistic for the analysis of biological microarray data J Bioinform Comput Biol 2005 3 1171 1189 10.1142/S0219720005001442 16278953

[B4] Breitling R Armengaud P Amtmann A Herzyk P Rank products: a simple, yet powerful, new method to detect differentially regulated genes in replicated microarray experiments FEBS Letters 2004 573 83 92 10.1016/j.febslet.2004.07.055 15327980

[B5] R http://www.R-project.org

[B6] R Development Core Team R: A language and environment for statistical computing 2005 R Foundation for Statistical Computing:. Vienna, Austria ISBN 3-900051-07-0.

[B7] Hong F Breitling R McEntee CW Wittner BS Nemhauser JL Chory J RankProd: A Bioconductor package for detecting differentially expressed genes in meta-analysis Bioinformatics 2006 22 2825 2827 10.1093/bioinformatics/btl476 16982708

[B8] GlaMA http://www.brc.dcs.gla.ac.uk/systems/glama/

[B9] haXe http://haxe.org/

[B10] Koziol JA Comment son the rank product method for analyzing replicated experiments FEBS Lett 2010 584 941 944 10.1016/j.febslet.2010.01.031 20093118 PMC2849678

