# Supplementary material for: RankProdIt: A web-interactive Rank Products analysis tool
Source: BMC Res Notes. 2010 Aug 6;3:221. doi: 10.1186/1756-0500-3-221 (PMC2930644; doi:10.1186/1756-0500-3-221)
Supplement: Additional file 3 — Description of columns in an output file. Gives a description of the contents of columns in a RankProdIt output file. [file 1756-0500-3-221-S3.PDF]

Description of columns in a RankProdIt output file

- **Gene_name:** contains the gene identifiers as selected by the user. Will always be the first column of the results file.
- **Cond1 columnXofInput:** contains data for one of the biological replicates of condition 1 submitted by the user, as contained in column X of the input. For every biological replicate of condition 1 a column of this type will appear. Only present in analyses of absolute level based data.
- **Cond2 columnXofInput:** contains data for one of the biological replicates of condition 2 submitted by the user, as contained in column X of the input. For every biological replicate of condition 2 a column of this type will appear. Only present in analyses of absolute level based data.
- **Cond1/Cond2 columnXofInput:** contains data for one of the biological replicates of condition 1/condition 2 submitted by the user, as contained in column X of the input. For every biological replicate of condition 1/condition 2 a column of this type will appear. Only present in analyses of ratio based data.
- **Cond2/Cond1 columnXofInput:** contains data for one of the biological replicates of condition 2/condition 1 submitted by the user, as contained in column X of the input. For every biological replicate of condition 2/condition 1 a column of this type will appear. Only present in analyses of ratio based data.
- **Cond1 < Cond2 rank:** contains the average rank of a gene when sorting data by condition 1 < condition 2 (based on Rank Products and/or Rank Sum analysis of RankProd [6]); genes with the highest average rank are the most down-regulated genes in

condition 1 compared to condition 2. Will always be present in the results file.

- **Cond1 > Cond2 rank:** contains the average rank of a gene when sorting data by condition 1 > condition 2 (based on Rank Products and/or Rank Sum analysis of RankProd [6]); genes with the highest average rank are the most up-regulated genes in condition 1 compared to condition 2. Will always be present in the results file.
- **Cond1 < Cond2 *p* value:** contains the *p* value of a gene (based on Rank Products and/or Rank Sum analysis of RankProd [6]) when sorting data by condition 1 < condition 2; genes with the lowest p. value are the most significantly down-regulated genes in condition 1 compared to condition 2. Will always be present in the results file.
- **Cond1 > Cond2 p. value:** contains the average rank of a gene when sorting data by condition 1 > condition 2 (based on Rank Products and/or Rank Sum analysis of RankProd [6]); genes with the lowest p. value are the most significantly up-regulated genes in condition 1 compared to condition 2. Will always be present in the results file.
- **Cond1 < Cond2 pfp value:** contains the probability of false prediction (pfp) of a gene when sorting data by condition 1 < condition 2 (based on Rank Products and/or Rank Sum analysis of RankProd[6]); genes with the lowest pfp value are the significantly most down-regulated genes in condition 1 compared to condition 2. Will always be present in the results file.
- **Cond1 > Cond2 pfp value:** contains the pfp of a gene when sorting data by condition 1 > condition 2 (based on Rank Products and/or Rank Sum analysis of RankProd[6]); genes with

the lowest pfp value are the most significantly up-regulated genes in condition 1 compared to condition 2. Will always be present in the results file.

- Average Cond1/Cond2: contains the average condition 1/condition 2 fold change on a linear scale. Only present in the results file if the user selects "linear scale" in the submission form and did not conduct an analysis of a condition 2/condition 1 experiment. Note that in data sets containing missing data the average is calculated by removing the missing data.
- Average Cond2/Cond1: contains the average condition 2/ condition 1 fold change on a linear scale. Only present in the results file if the user selects "linear scale" in the submission form and conducted analysis of a condition 2/condition 1 experiment. Note that in data sets containing missing data the average is calculated by removing the missing data.
- Log(2) Average Cond1/Cond2: contains the average condition 1/condition 2 fold change on a log_2 scale. Only present in the results file if the user selects "log(2) scale" in the submission form and did not conduct an analysis of condition 2/condition 1 experiment. Note that in data sets containing missing data the average is calculated by removing the missing data.
- Log(2) Average Cond2/Cond1: contains the average condition 2/condition 1 fold change on a log_2 scale. Only present in the results file if the user selects "log(2) scale" in the submission form and conducted analysis of a condition 2/condition 1 experiment. Note that in data sets containing missing data the average is calculated by removing the missing data.
